# Recent Synthesis Routes to Previously Inaccessible Zeolite Porosity: From Ultrasmall Pore to Ultraporous Materials

**DOI:** 10.1021/acsami.6c06152

**Published:** 2026-06-18

**Authors:** Jiho Shin, Huajian Yu, Donghui Jo, Zihao Rei Gao, Luis A. Villaescusa, Suk Bong Hong, Miguel A. Camblor

**Affiliations:** † Green Carbon Research Center, 65680 Korea Research Institute of Chemical Technology (KRICT), 141 Gajeong-Ro, Yuseong-gu, Daejeon 34114, Korea; ‡ 69570 Instituto de Ciencia de Materiales de Madrid (ICMM), CSIC, C/Sor Juana Ines de la Cruz, 3, 28049 Madrid, Spain; § Separation & Purification Research Center, Korea Research Institute of Chemical Technology (KRICT), 141 Gajeong-ro, Yuseong-gu, Daejeon 34114, Korea; ∥ Department of Chemical and Biomolecular Engineering and Institute for NanoBioTechnology, Johns Hopkins University, 3400 N Charles Street, Baltimore, Maryland 21218, United States; ⊥ Instituto Interuniversitario de Investigación de Reconocimiento Molecular y Desarrollo Tecnológico (IDM) Universitat de València−Universitat Politècnica de València, Camí de Vera s/n, 46022 Valencia, Spain; # CIBER de Bioingeniería Biomateriales y Nanomedicina (CIBER-BBN), Camí de Vera s/n, 28029 Madrid, Spain; ¶ Departamento de Química, Universitat Politècnica de València, Camí de Vera s/n, 46022 Valencia, Spain; ∇ International Center for Ordered Nanoporous Materials Synthesis, Shanxi Research Institute of Huairou Laboratory, Taiyuan 030032, P. R. China

**Keywords:** zeolite synthesis, structure-direction, unconventional synthesis, ultraporous zeolites, open small pore zeolites

## Abstract

The diverse porosity and properties of zeolites, together with their stability, drive applications from catalysis to adsorption. Conventional hydrothermal synthesis has yielded a large number of zeolite framework types, the vast majority of which are synthetic. However, this approach still has limitations, as exemplified by the large disparity between known and hypothetical but feasible framework types. This Perspective highlights recent unconventional synthesis routes that expand the range of attainable structures and porosities: ultrasmall open pores by zeolite reconstruction; unfeasible zeolites with tunable pores by assembly disassembly organization-reassembly; the embedded isoreticular RHO zeolite family synthesized using mixed inorganic cations; shape selective small and medium pore zeolites synthesized via an excess fluoride approach; and phosphonium-enabled ultraporous zeolites (<13.2 tetrahedral atoms/1000 Å^3^). Their associated applications include selective dehydration, CO_2_ capture, catalysis, and decontamination. Although challenges such as scalability, organic structure-directing agent cost and emissions persist, these unconventional chemistries offer important opportunities for further evolution of zeolite structures for environmental, energy and health-related applications.

## Graphical abstract

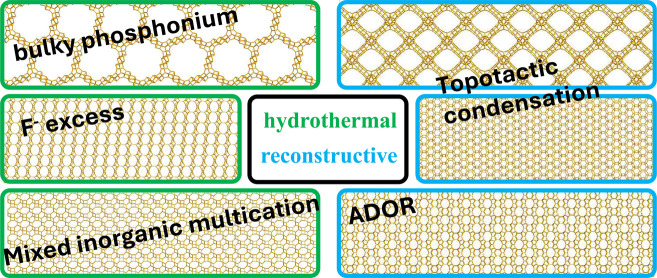

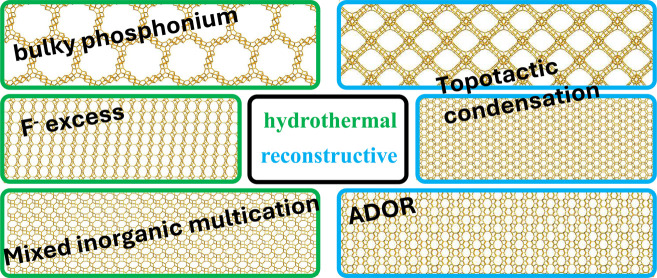

## Introduction

Zeolites are crystalline, microporous materials with three-dimensional (3D) structures containing well-defined channels and cavities of molecular dimensions, which confer exceptional properties in adsorption, ion-exchange, and, when provided with active sites, catalysis. The unique combination of high internal surface area, well-defined porosity, catalytically active sites (such tunable Brönsted and Lewis acid sites), and robust thermal and chemical stability has made zeolites invaluable in heterogeneous catalysis, particularly in petroleum refining and petrochemical transformations, where they replaced corrosive mineral acids, facilitating catalyst recovery and enabling energy- and atom-efficient processes.^1,2^ The improved selectivity of zeolitic catalysts results in minimal formation of undesired byproducts, thereby reducing waste generation, lowering raw material consumption, and lessening the environmental and economic burden associated with handling and disposing of side products. Beyond industrial catalysis,^3^ zeolites also play a central role in gas separation,^4,5^ water purification,^6,7^ detergency,^8^ waste treatment,^9,10^ environmental decontamination,^11,12^ and in emerging applications in energy,^13,14^ high temperature superconductivity,^15^ and biomedical technologies.^16,17^

The search for new zeolite structures that could deliver new or improved applications has typically relied on our limited and largely empirical knowledge of structure-direction effects, that is, the factors determining which zeolite crystallizes under given conditions.^18^ Despite its empirical character, such an approach has been remarkably successful, as illustrated by the large number of synthetic zeolites phases currently known. The International Zeolite Association currently recognizes 265 unique zeolite framework types (ZFTs) that have been obtained as ordered crystalline materials, together with 42 families of disordered materials consisting of intergrowths of different structure types, some of which have never been obtained as pure, ordered polymorphs and therefore do not count among the 265 ZFTs.^19^ The zeolite structure landscape is now clearly dominated by synthetic materials: over 80% of the known framework types can be obtained in the lab, whereas only a relatively small fraction, less than one-fourth, occur naturally. Synthetic efforts have enabled not only the discovery of most zeolite structures but also a broad compositional variability that has considerably expanded their applications, since composition strongly influences adsorption and catalytic performance.

The limitations related to the noted large empirical character of zeolite discovery and the incomplete understanding of structure-direction relationships may be at the core of the large disparity between the scarce number of known ZFT and the very large number of hypothetical frameworks.^20–22^ This disparity becomes even more striking when the energetics of the hypothetical frameworks are considered, since a huge number of them are close in energy to the values observed in known zeolites and, hence should be considered “feasible”.^21^ This lack of fundamental knowledge is a strong handicap when targeting specific porosity regimes. In particular, conventional hydrothermal synthesis has so far failed to provide open ultrasmall-pore and fully connected ultraporous frameworks. In our view, the constraints of our current knowledge of structure-direction can motivate the exploration of alternative synthesis concepts that expand the accessible ‘porosity space’ beyond what is commonly obtained under standard hydrothermal conditions.

Historically, the discovery of zeolites A (LTA) and X and Y (FAU) in the midtwentieth century,^23^ together with the recognition of their distinctive properties,^24^ was quickly followed by major commercial applications.^25^ The ensuing continuous feed-back between zeolite synthesis and zeolite applications has driven the field forward with remarkable momentum. Currently, a wide variety of zeolites, in terms of structures, compositions and textural properties provides a high degree of tunability to target specific applications.^26^ At the same time, although zeolite synthesis has long been dominated by hydrothermal routes in which structure-directing effects are exploited and screened to discover new materials, multiple alternative synthesis strategies have emerged over the last few decades, including topotactic condensation of layered and chain silicates, interunit expansion of such precursors, recrystallization and reconstruction processes such as assembly disassembly organization-reassembly (ADOR) and ionothermal synthesis.

Structural porosity is the most characteristic and defining feature of zeolites, although their notable chemical and (hydro)­thermal stability, together with the possibility of introducing functionality (for example, acid sites in H^+^-exchanged aluminosilicate zeolites or metal centers at framework positions via isomorphous substitution), is equally crucial for their applicability. Zeolite pores are typically classified based on the size of the smallest window along the diffusion path, expressed as the number of tetrahedra forming that window, into small (8 Si–O tetrahedra, 8R) medium (10R), large (12R) and extra-large (>12R). “Odd zeolites”, that is, zeolites with odd numbered pores (3R, 7R, 9R, 11R, 15R, 21R) are also known but are exceedingly rare, with less than 5% of all known ZFTs showing odd pores,^27^ and the reasons for this scarcity remain unclear.^28^ In retrospect, it is evident that zeolites with very different pore sizes may find useful applications, which rationalizes the sustained efforts devoted to synthesizing new structures: the broad diversity of pore architectures enables fine-tuning when selecting materials for a given function. For instance, FAU type zeolites, with a 3D system of large pores, are widely used in catalysis (for example in fluid catalytic cracking, FCC), whereas the small pore aluminosilicate LTA with a Si/Al ratio of unity excels in cation-exchange applications.^29^ Medium pore zeolites, which display strong shape selectivity effects,^30^ are extensibly applied in catalysis for petrochemical and refinery processes,^31–33^ and in the synthesis of fine chemicals.^26,34–36^ And small pore zeolites are central to ammonia selective catalytic reduction, methanol-to-olefins conversion, methane oxidation, and methylamines synthesis.^37^

Figure 1 shows the distribution of ZFTs as a function of framework density FD_Si_ (defined as the number of tetrahedral atoms, T atoms, per 1000 Å^3^ in an idealized SiO_2_ structure in the highest topological symmetry of each ZFT) and compares the current histogram with that existing in the sixth revised edition of the Atlas of Zeolite Framework Types (2007).^38^ In addition to a 50% increase in the number of ZFTs in less than 20 years, the histogram has changed shape: the relative increase in ZFTs on the low density side (<13 T atoms/1000 Å^3^) is larger than that observed in other FD_Si_ ranges. This trend signals the recent success of the efforts aimed at generating zeolites with very open frameworks.

**Figure 1:**
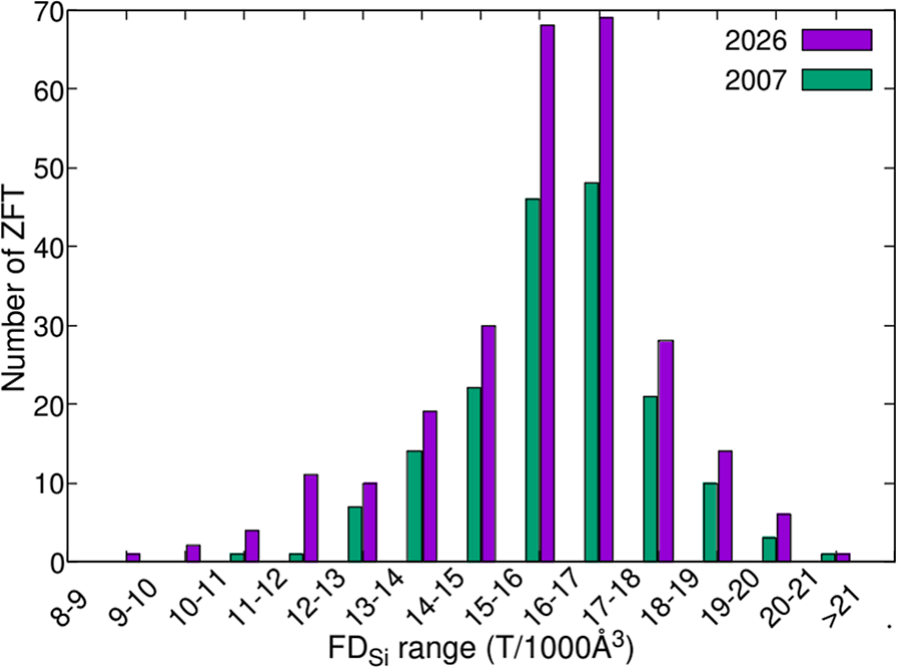
Comparison of Zeolite Framework Types (ZFTs) classified by FD_Si_ range in 2007 and 2026, highlighting the significant expansion of new zeolite structures developed over the past two decades.

This perspective offers a panoramic and forward-looking view of the development of zeolite synthesis, focusing on recent unconventional preparation routes, which here refer to unconventional chemistries rather than to unconventional physical conditions.^39^ Although the whole spectrum of zeolite porosity is addressed, particular emphasis is placed in both ends of the porosity range (open ultrasmall and ultraporous materials) and on the new opportunities that these unconventional routes create for sustainable applications, from environmental catalysis to energy. Apart from the ADOR process, other top-down strategies for generating hierarchical or mesoporous zeolites fall outside the scope of this work, and the interested reader is referred to a recent review devoted to such methods.^40^

### Open Ultrasmall Pore Zeolites by Topotactic Condensation and by Reconstruction

Zeolites with 6R “pores” have traditionally been regarded as nonporous, although they still qualify as zeolites when relatively large internal cavities lead to low framework densities, as in clathrasils. The presence of inorganic cations in low-silica zeolites and organic cations in high-silica zeolites as guest species apparently renders 6R-pore materials unusable as adsorbents. Nevertheless, cation exchange in the 6R sodalite (SOD) zeolites from aqueous solutions has been long known to occur,^41,42^ opening possibilities for cation exchange applications, such as the sequestration of radioactive cations by low-silica (Si/Al < 2) 6R zeolites. In contrast, gas adsorption applications of high-silica 6R zeolites require the removal of organic structure-directing agents (OSDAs), which are typically trapped inside during synthesis, and are difficult to remove.

This challenge was overcome by a prolonged calcination treatment: a 7 day calcination at 680 °C in N_2_ for ethylene glycol-containing pure-silica SOD in N_2_, followed by an equally long (7–14 days) calcination in air at the same temperature.^43^ The resulting rhombohedral SiO_2_ polymorph preserves the SOD framework and can adsorb disulfur dinitride (S_2_N_2_) vapor, likely after decomposition of this square planar molecule. In our view, the success of the ethylene glycol removal from pure-silica SOD is due to the long initial calcination in N_2_, which avoids the need for O_2_ to diffuse into, and combustion products to diffuse out of, the zeolite through the small 6R windows, and may also be facilitated by the very high flexibility of the SOD framework.^44,45^ Although effective, such harsh conditions are hardly practical as a general route to open 6R pores.

More relevant for the purpose of this perspective are two synthetic approaches that generate open 6R pores by alternative routes avoiding extreme calcination protocols. Pure-silica SOD without guest species was reported by Moteki et al., who obtained it by topotactic condensation of a layered precursor, RUB-15, previously treated with acetic acid to remove the organic cation used as OSDA while preserving the layer ordering, followed by calcination at 800 °C. The resulting SOD was able to adsorb H_2_, demonstrating the accessibility of its 6R pores.^46^ Later, Koike et al. condensed the same acetic-acid-treated layered precursor by refluxing in *N*-methylformamide at 180 °C and subsequent calcination at 800 °C, affording pure-silica SOD with a reportedly higher quality.^47^

An even more striking example is provided by a very recent report that achieves the goal of open 6R pores at substantially lower temperatures through a reconstructive route.^48^ First, the aluminophosphate molecular sieve ZJM-8 (JSY) was synthesized hydrothermally at 200 °C using 1,4-hexaethylbutane-1,4-diammonium as OSDA. ZJM-8 belongs to the ABC-6 family, has a 10-layer stacking sequence AABAACCBCC­(A) along the *c* axis, exhibits a 3D system of 8R pores, contains abundant double 6R (*d6r*) units, and readily adsorbs N_2_. Then, the calcined ZJM-8 was treated in water at room temperature and then calcined at 550 °C, suffering a reconstructive transformation involving cleavage of certain interlayer connections, layer displacement, and subsequent reconnection, yielding ZJM-9 (ZJN). ZJM-9 also belongs to the ABC-6 family but adopts a different ten-layer stacking sequence, CABACACBCA­(C), due to the absence of *d6rs*, and contains a 3D system of 6R pores. ZJM-9 does not adsorb N_2_ but does adsorb water. It displays a large selectivity for water in mixtures with CH_3_OH, CO, CO_2_, and CH_4_, similar to that of commercial 3A zeolite, but with a much lower temperature for water desorption (95 °C compared to >250 °C), which highlights its potential as an adsorbent for gas dehydration.

Thus, beyond extreme and costly calcination protocols, the fundamental challenge posed by OSDAs irretrievably trapped in 6R pores during synthesis can be addressed through approaches in which organic species are removed prior to topotactic condensation of layered precursors or are eliminated during a reconstructive transformation, so that the final materials display open 6R pores accessible to small molecules. These strategies convert nominally “nonporous” small-pore zeolites into functional adsorbents, thereby demonstrating that 6R-based frameworks can support practical separation and dehydration applications when their intrinsic pore systems are unlocked.

### Zeolites by the ADOR Process

ADOR is a controllable approach to postsynthesize daughter zeolites with better stability than the parent ones, utilizing the vulnerability of Ge-rich *d4r*s, which can be easily hydrolyzed. Daughter zeolites typically retain the structural integrity of the layers originally connected through *d4r*s but exhibit equal or smaller pore sizes, depending on the final interlayer connections (*d4r*s, single 4R (*s4r*s), or O bridges). This process is considered as a strategy for the formation of “unfeasible structures” that cannot be obtained from conventional hydrothermal synthesis.^49^ The first case of ADOR was reported by Roth et al. in 2011 using germanosilicate UTL as a parent zeolite.^50^ After hydrolysis of the interlayer Ge-rich *d4r*s, the extra-large pore (ELP) UTL transformed into layered IPC-1P, which is the precursor of most of the IPC series materials. Direct calcination of IPC-1P leads to poorly ordered IPC-1 via layer condensation, while the ordered zeolite IPC-4 (PCR) is prepared by the condensation of IPC-1P after intercalation with octylamine.^51^ Alternatively, IPC-1P can be converted into IPC-2 (OKO) through silylation with diethoxydimethylsilane followed by calcination.^50^ These results suggest that the layers are poorly aligned after hydrolysis, while the intercalation and silylation can promote their organization.

Interestingly, intercalation with OSDA-type organics (e.g., octylamine) or reaction with alkoxysilanes (e.g., diethoxydimethylsilane) is not necessary for the organization step in ADOR. A self-organization of the IPC-1P layers can occur upon heating under certain acidic conditions.^52,53^ The hydrolysis of Ge,Si-UTL under highly acidic conditions (>7 M HCl) and heating involves Si rearrangement and/or migration into *s4r* precursors from *d*4*r* and/or interlayer. Subsequent calcination leads to IPC-2 by condensing *s4r* and IPC-1P layers. Another derivative of OKO is COK-14, which is obtained by acid treatment in 12 M HCl solution followed by subsequent calcination.^54^ An intermediate phase Ge-COK-14, with Ge-4Rs accommodated in the channels, was captured by Verheyen et al. in HCl solution of the same concentration for a shorter time. These processes can be considered as the inverse sigma transformation of UTL through the removal of a layer of framework atoms and subsequent contraction.

However, hydrolysis and heating under neutral or low acidic conditions (neutral to 0.1 M HCl) only removes *d4r*s, leading to the formation of IPC-4 upon calcination. After acid treatment at intermediate concentrations and subsequent calcination, IPC-1P condenses to two other materials (IPC-6 and IPC-7). When the rates of rearrangement and deintercalation are equal, IPC-6 is formed with the same level of *s4r* connection and oxygen bridging. Under higher acidity, the resulting material IPC-7 possesses an equal quantity of *s4r* and *d4r* connecting the layers after calcination. The cases from IPC-1P to IPC-4, IPC-6, IPC-2, and IPC-7 demonstrate that ADOR is a promising process to manipulate zeolites with continuously tunable porosity from UTL by adjusting the hydrolysis conditions (Figure 2).^53^ Interestingly, instead of achieving good alignment like its parent material, forcing a small geometric mismatch can be an approach to synthesize new zeolites as well. For instance, the incorporation of choline cations in the ADOR process can induce a relative shift between IPC-1P layers, leading to the formation of IPC-9P. Calcination of IPC-9P, either directly or following intercalation with diethoxydimethylsilane, yields IPC-9 and IPC-10, respectively.^49^

**Figure 2:**
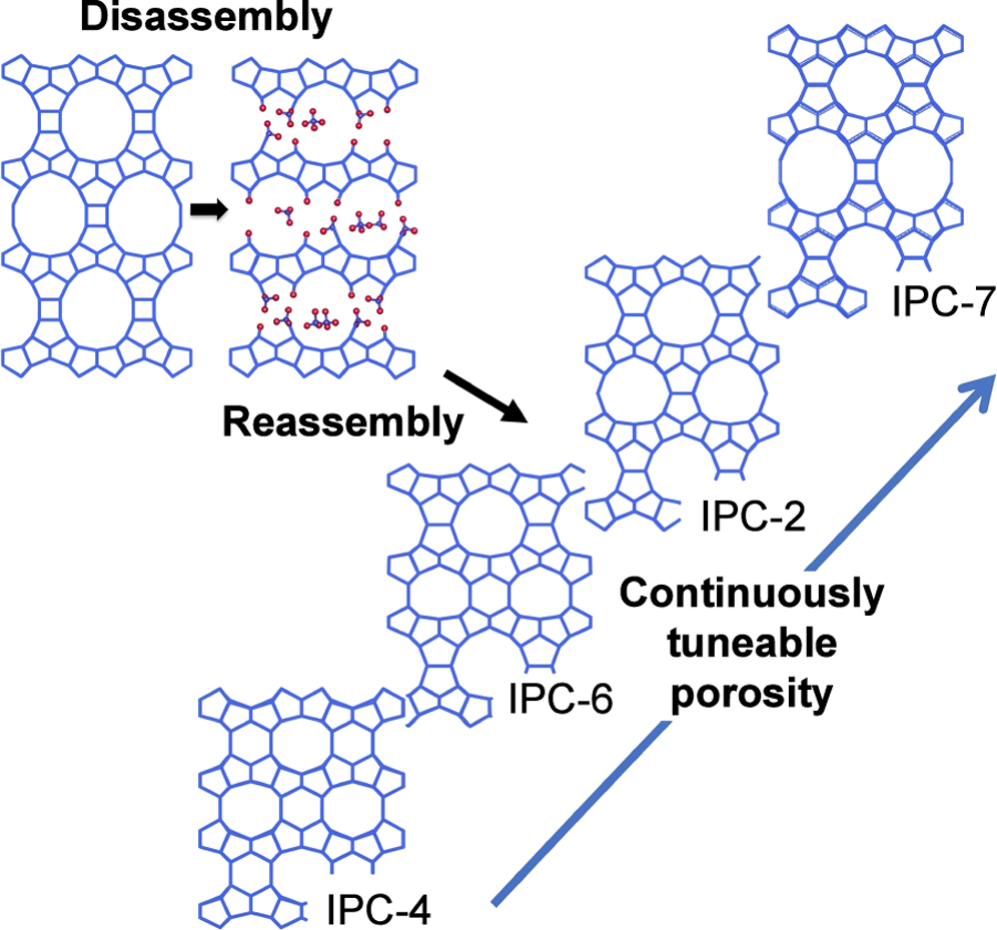
Porosity of zeolites generated by ADOR (from Ge,Si-UTL to IPC-1P, IPC-2, IPC-4, IPC-6, and IPC-7). Adapted with permission from ref 53. Copyright 2014 Wiley-VCH.

In addition to Ge,Si-UTL, Ge, Si zeolites with other frameworks topologies like UOV, CIT-13, IWW, IWV and IWR have been successfully used in the ADOR process to discover new structures.^52,55^ Zeolites amenable to ADOR are known as *ADORable*. IPC-12 is synthesized from UOV by removing *d4r*s for direct oxygen connections.^56^ The transformation only occurs in a single direction, which is perpendicular to the 12-/8- channels of UOV. From SAZ-1 (isostructural to CIT-13), two zeolites (designated as IPC-15 and IPC-16) are obtained by intercalation with octylamine and reaction with diethoxydimethylsilane before calcination, respectively.^57^ The hydrolysis of Ge-rich IWW results in IPC-5P, which can be restored to IWW by reaction with diethoxydimethylsilane.^58^ Another derivative of IWW is IPC-18, which was prepared by vapor-phase-transport (VPT) arrangement using 12 M HCl solution.^59^ During the VPT treatment, the hydrolysis and rearrangement of Ge-rich units of IWW lead to the transformation of *d4r*s to *s4r*s, resulting in IPC-18 with a shorter interlayer space. Recently, IPC-20 was obtained from IWV by controlling the structure disassembly and the interlayer hydrolysis rates using MeOH/H_2_O solution followed by calcination.^60^ IPC-20 features *s4r*s derived from the original *d4r*s in its parent IWV, while preserving the layer structure. On the other hand, many germanosilicates with *d4r*s, for instance, IWR, ITR, and ITH, are considered in principle as *ADORable* zeolites. However, the inappropriate chemical compositions of these zeolites hamper the success of the whole ADOR processes, although their disassembling into layered materials could be realized.^61^ In fact, by optimizing the chemical compositions and crystallite dimensions of the parent IWR in the assembly step, IPC-17 was synthesized by hydrolysis (using concentrated HCl aqueous solution or VPT with HCl vapor) and subsequent condensation at elevated temperatures.^55^

The ADOR process provides continuous control of the porosity of zeolites via gradual interlayer hydrolysis and reconnection, which is important for gas adsorption and storage. For instance, IPC-12 and IPC-17 (derived from UOV and IWR, respectively) are considered as efficient adsorbents for CO_2_ due to their structural motifs and/or pore systems.^55,62^ With tunable interlayer spacing and intact intralayer structures, in addition, zeolites derived from ADOR open new possibilities in shape-selective catalysis. IPC-18 offers high conversion and selectivity in the hydration of ethylene oxide to ethylene glycol due to the presence of acid sites and appropriate pore size in its framework.^63^ Metal species can be encapsulated in zeolites by first inserting them within the layered materials followed by subsequent condensation of the layers via calcination. Pt@IPC-2 and Pt@IPC-4 were synthesized from the IPC-1P layered precursor via calcination, which was preceded by silane intercalation for Pt@IPC-2 and swelling for Pt@IPC-4, respectively.^64^ Li et al. employed surfactants of various lengths and a Rh source for swelling to introduce Rh within the interlayer space of IPC-1P.^65^ Upon calcination, Rh nanoparticles can be encapsulated within the resulting disordered mesoporous material, Rh@IPC_C22, exhibiting high resistance to Rh sintering at 750 °C due to the strong interaction between the Rh nanoparticles and the surface silanol quadruplets of IPC-1P.

Here we propose that ADOR could in principle be used as an alternative to produce zeolites with open ultrasmall pores, provided that in 8R and 10R zeolites with dense layers separated by *d4r*s these units could be replaced by O–Si–O and simple O connections, respectively. The germanosilicate 10R zeolite PST-35 (PTF) could be a potential starting material for this process (Figure 3).

**Figure 3:**
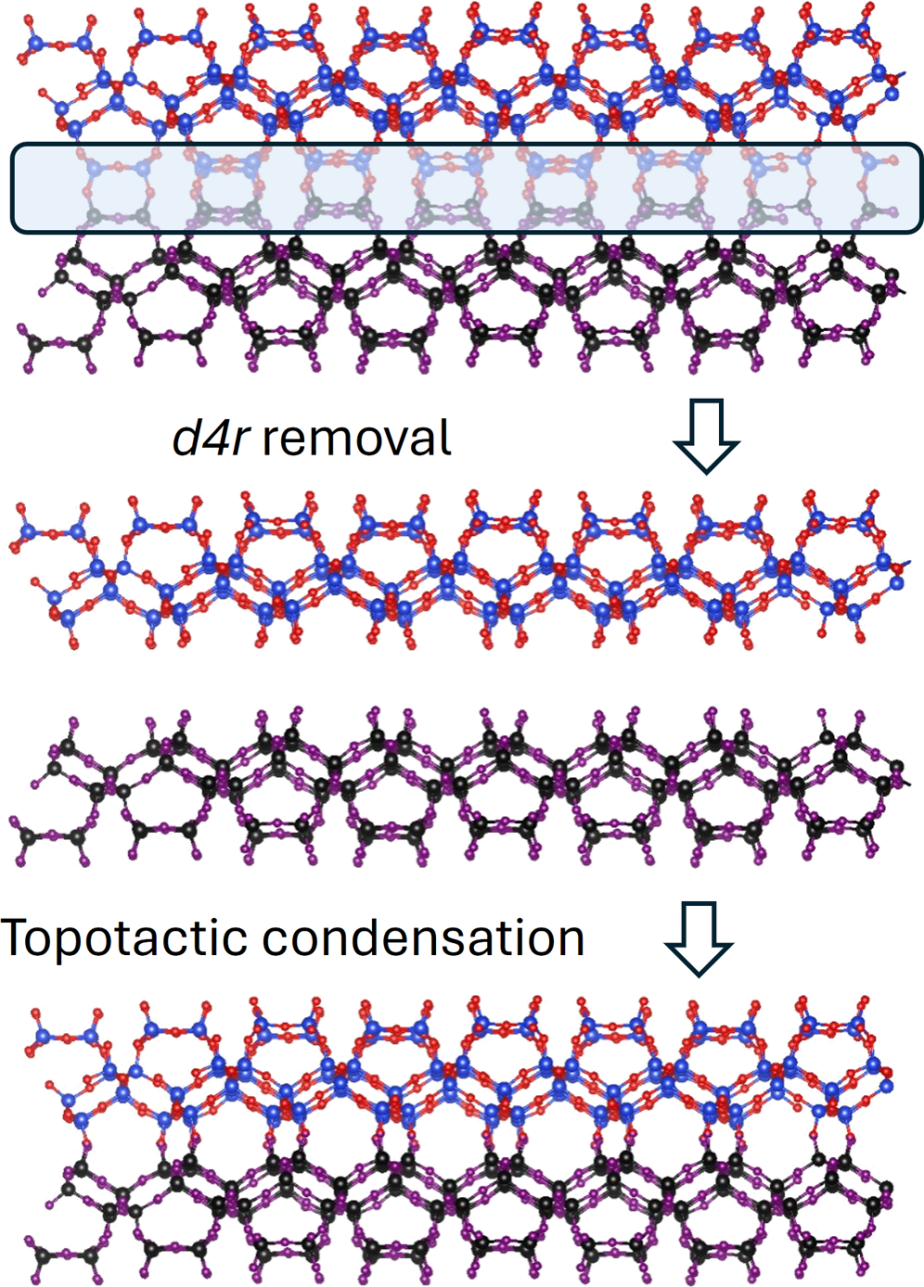
A zeolite with open ultrasmall 6R pores could be hypothetically obtained by an ADOR process on Ge,Si-PST-35 (top). Elimination of *d4rs* in the PST-35 structure (highlighted rectangle) by an acid treatment would produce a layered material (middle) which by topotactic condensation would result in an open ultrasmall 6R pore zeolite (bottom). The layers are colored differently for the sake of clarity: Si and O atoms are blue and red, respectively in the upper layer and black and purple, respectively, in the bottom one.

### Multiple Inorganic Cation Approach (Embedded Isoreticular Zeolite Family and Other Novel Zeolites)

Zeolite synthesis has historically been guided by the design of OSDAs which have served as the principal means of controlling framework topology and pore architecture. Within this OSDA-centered paradigm, inorganic structure-directing agents (ISDAs) like alkali and alkaline-earth metal ions have often been regarded primarily as charge-balancing species for compensating framework negative charges produced by Al-substitution. However, changes in the type and concentration of ISDAs have frequently led to distinct crystallization outcomes even in the presence of identical OSDAs, suggesting that the product selectivity is not governed solely by OSDA design,^66–68^ which challenges the conventional view of inorganic cations as passive counterions and instead reveals their important role in directing zeolite crystallization pathways. ISDAs exhibit intrinsic differences in the hydration behavior, ionic radius, and charge density, depending on their type, influencing the assembly of secondary building units and cation–framework interactions during nucleation and solubility of specific (or not) nutrients.^69–71^ In addition, they can participate in electrostatic and coordination interactions with OSDAs, thereby influencing the locations and conformations of OSDAs occluded within the zeolite pores.^72–76^ Apparently, when more than one type of ISDAs exists in zeolite synthesis mixtures, the resulting interactions with OSDAs might operate cooperatively or competitively, thereby altering crystallization pathways. Such mixed environments can selectively promote the formation of specific secondary building units or modify nucleation barriers, thereby expanding the range of accessible framework structures. From this perspective, the multiple inorganic cation approach is best understood not as a simple variation in counterion type, but as the deliberate engineering of cooperative organic–inorganic interactions that govern zeolite nucleation and crystal growth.^77^

One of the most representative examples of the multiple inorganic cation approach as a design principle is found in the RHO family of embedded isoreticular zeolites (EIZ),^78,79^ where the cooperative incorporation of distinct ISDAs directs specific building unit formation and increases structural complexity. Elucidation of the structural relationships among the RHO, PAU, and MWF frameworks revealed a stepwise expansion mechanism in which successive insertion of *d8r* and *pau* cages between *lta*-based scaffolds results in an approximately 10 Å increase in unit-cell dimension per generation (Figure 4a). The interscaffold space is concurrently filled with embedded cages such as *t-plg*, *t-gsm*, *t-oto*, and *t-phi*, thereby preserving the maximum ring size while progressively increasing structural complexity across generations. Within this family, zeolite RHO represents the first generation (RHO-G1), while ECR-18 (PAU), a synthetic analogue of paulingite, and ZSM-25 (MWF) correspond to the third (RHO-G3) and fourth (RHO-G4) generations, respectively. Guided by this framework expansion principle, higher-generation EIZ structures beyond ZSM-25, corresponding to RHO-G5 and RHO-G6, were predicted. Experimental realization of these higher generations was achieved by intentionally introducing alkaline-earth cations (Ca^2+^ or Sr^2+^) into the ZSM-25 synthesis system containing tetraethylammonium (TEA^+^) and Na^+^ ions, yielding PST-20 (RHO-G5) and PST-25 (RHO-G6).^78^ Unlike synthesis systems containing only Na^+^ ions, the additional alkaline-earth cations favored the formation of embedded cages such as *t-gsm*, *t-oto*, and *t-phi*, thereby enabling progressive framework expansion toward higher-generation EIZ structures. Further adjustment of inorganic synthesis parameters, particularly both the Al and H_2_O contents in the synthesis mixture, allowed access to the more complex generations PST-26 (RHO-G7) and PST-28 (RHO-G8).^79^ Notably, some higher-generation EIZs such as ZSM-25 and PST-20 have also shown promising CO_2_ adsorption and separation performance, demonstrating the functional potential of these increasingly complex framework architectures.^80^

**Figure 4:**
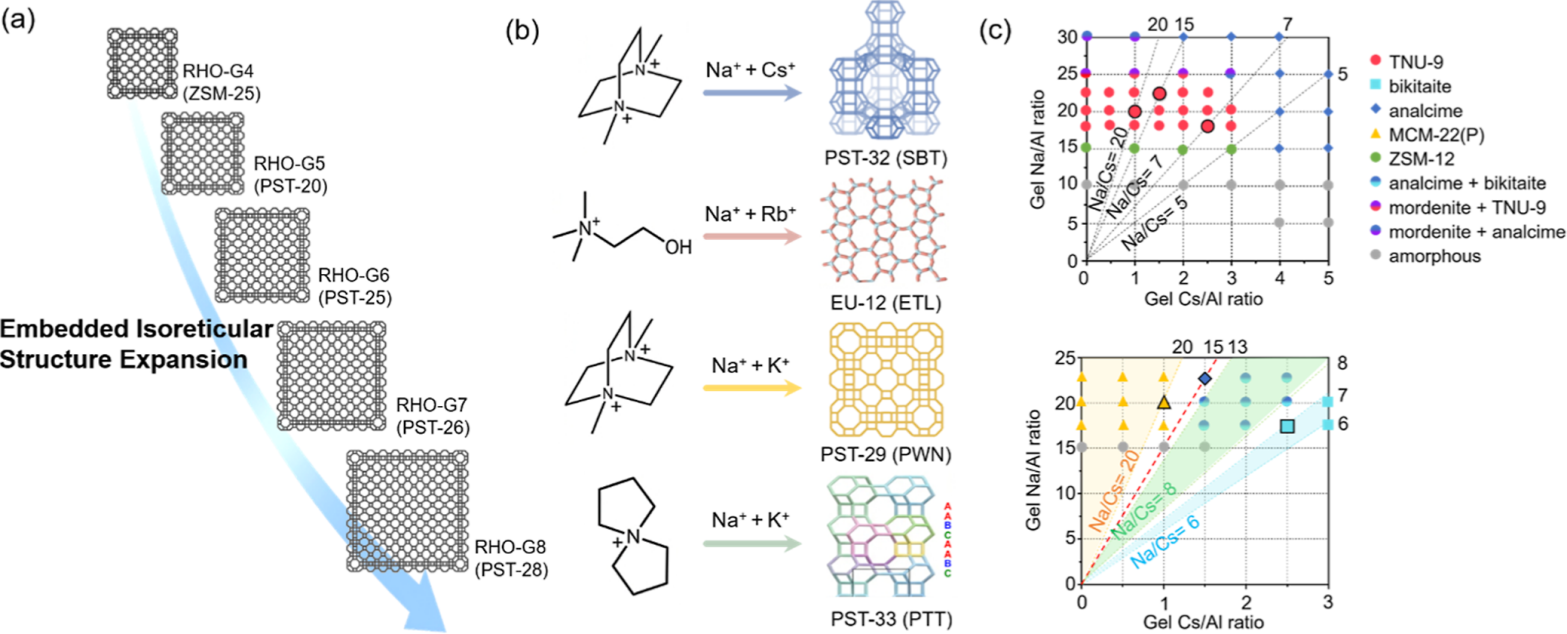
(a) Representative cross-sectional views (ca. 12 Å thick) illustrating the progressive structural expansion within the RHO family of EIZs. The higher generations (RHO-G5 to RHO-G8) were obtained by introducing alkaline earth cations (e.g., Ca^2+^ or Sr^2+^) into the ZSM-25 (RHO-G4) synthesis mixture, which facilitated the formation of embedded *t-gsm*, *t-oto*, and *t-phi* cages required for further structural expansion. Adapted with permission from ref 79. Copyright 2016 Wiley-VCH. (b) Representative examples of aluminosilicate zeolites with new framework structures and/or compositions synthesized via a multiple inorganic cation approach. Cooperative structure direction between OSDAs and more than one type of ISDAs enabled access to those that were not attainable in the presence of one type of ISDAs. (c) Solid products isolated during TNU-9 synthesis at 150 °C using gels containing 1,4-bis­(*N*-methylpyrrolidinium)­butane as an OSDA with identical Si/Al ratios (50) but different Na/Al and Cs/Al ratios. The top panel summarizes the final products isolated after 21 days, whereas the bottom one shows the intermediate phases obtained after 4 days of crystallization. Mixed-phase samples are represented by split symbols, and amorphous products remained unchanged for up to 21 days of heating. Adapted with permission from ref 86. Copyright 2024 American Chemical Society.

Beyond progressive framework expansion, cooperative inorganic cation effects can enable the emergence of entirely new zeolite structures and compositions by altering building unit formation and assembly pathways during crystallization. Individual inorganic cations can each preferentially stabilize different building units through their distinct interactions with OSDAs and aluminosilicate species, whereas cooperative interactions among multiple inorganic cations may give rise to entirely new building units that are inaccessible under single-cation conditions. Moreover, these effects can further lead to previously inaccessible modes of framework assembly and connectivity during crystallization, thereby opening new routes toward novel framework structures and compositions. This principle is well demonstrated by the synthesis of PST-32 (SBT) and the related PST-34 family of FAU/SBT/SBS intergrowths using mixed Na^+^ and Cs^+^ inorganic cation environments in the presence of *N*,*N′*-dimethyl-1,4-diazabicyclo­[2.2.2]­octane (Me_2_-DABCO) as a relatively unselective OSDA (Figure 4b).^81,82^ PST-32 is the thermally stable aluminosilicate version of the cage-based large-pore zeolite SBT, which had previously been synthesized only in phosphate-based compositions. In this system, the cooperative effect of Na^+^ and Cs^+^ promoted the formation of *can* cages as basic building units and the connection of *can* layers along the *c*-axis, thereby enabling the crystallization of the SBT zeolite in industrially relevant aluminosilicate compositions.^81^ Under closely related synthesis conditions, fine control of the Na^+^/Cs^+^ ratio instead led to the synthesis of PST-34 intergrowth phases composed of vertically coupled *sod* and *can* cage layers. These phases exhibit systematically tunable FAU fractions, yielding FAU/SBT/SBS intergrowth phases with different layer-stacking sequences. In the latter case, Na^+^ and Cs^+^ exhibit complementary preferences for *sod* and *can* cage formation, respectively, and their cooperative interactions govern layer stacking and phase competition.^82^ Similarly, cooperative effects between different inorganic cations have also been observed in directing the synthesis of zeolites with novel framework structures, including EU-12,^83^ PST-29,^84^ and PST-33,^85^ where crystallization occurred only within narrow mixed-cation compositional windows (Figure 4b). Although the precise crystallographic locations of the inorganic cations could not be fully resolved in these systems, precluding detailed identification of their specific structure-directing roles, these results nevertheless suggest that cooperative inorganic cation effects play an important role in enabling crystallization pathways that are inaccessible under single-cation conditions.

Cooperative ISDA effects are not limited to altering phase selectivity but can also fundamentally alter crystallization kinetics and phase transformation pathways. During the synthesis of TNU-9 (TUN), systematic variation of the Na^+^/Cs^+^ ratio in the gel stabilized different crystalline intermediate phases prior to formation of the final product, including high-silica versions of small-pore zeolites bikitaite (BIK), and analcime (ANA), as well as the layered MWW precursor (Figure 4c).^86^ These results demonstrate that such effects can modulate the nucleation sequence and intermediate phase evolution. Mallette et al. reported that, in the synthesis of CHA zeolite, the combination of inorganic cations with contrasting hydration properties markedly shortens the nucleation period and overall crystallization time, suppresses competing metastable phases such as FAU, and expands the temperature window for CHA formation, thereby enhancing crystallization efficiency while simultaneously enabling control over crystal size and defect density.^87^ These studies establish that cooperative inorganic cation chemistry influences both kinetics and thermodynamics across all stages of zeolite crystallization.

Overall, the introduction of alkali or alkaline-earth metal ions as a second ISDA can serve as an architect that reconfigures organic–inorganic synergy under crystallization conditions. Through precise control of cation combinations and ratios, it becomes possible to systematically tailor framework topology, composition, crystallization kinetics, and intermediate phase selectivity, which holds significant industrial relevance. However, the enormous combinatorial space defined by cooperative OSDAs and engineered mixed inorganic cation environments remains largely unexplored. Recent advances in computational modeling and machine learning have the potential to facilitate more systematic analysis of the cooperative structure-directing effect of ISDAs during zeolite crystallization.^72,88–92^ Clarifying how engineered cation combinations, in concert with specific organic molecules, bias secondary building unit formation and nucleation pathways could provide more rational access to previously underexplored zeolite structures and compositions.

### Excess Fluoride Approach

The work by Camblor and co-workers in the late 1990s, in which highly concentrated fluoride media (typically with H_2_O/SiO_2_ < 10 and HF/OSDA^*q*+^(OH)_*q*_ = 1.0) were applied in zeolite synthesis, resulted in the observation of the Villaescusa’s rule–when one particular OSDA can direct the synthesis of several different zeolite phases, the structure with a lower framework density tends to form under more concentrated conditions^18^–which still lacks sufficient understanding of its mechanism. This concentrated synthesis route also led to the discovery of a number of new zeolite structures,^93^ and its validity was first demonstrated at pure-silica composition using TEA^+^ as an OSDA.^94,95^ Although zeolite beta crystallized over a broad range of HF/SiO_2_ ratios (0.3–2.0), the crystallization yield was maximized and the crystallization time minimized around HF/SiO_2_ = 0.5, i.e., at an HF/TEAOH ratio of unity. Consequently, the simultaneous use of equimolar concentrations of HF and OSDA^+^ under highly concentrated conditions has proven highly effective for finding many novel pure-silica zeolites like ITQ-3 (ITE), ITQ-7 (ISV), ITQ-12 (ITW), and ITQ-13 (ITH).^93,95^

On the other hand, only intermittent efforts have been made to identify the effect of HF concentration on zeolite synthesis. In 1996, Cheetham et al. varied the amount of HF in the HF/pyridine/propylamine system (HF/pyridine ratio from 2.0 to 6.0); however, the resulting phase was exclusively pure-silica ferrierite (FER), with larger crystals formed at higher HF concentrations.^96^ In 2016, Zhang et al. claimed that pyrrolidinium-based OSDAs and TEA^+^ under high HF concentrations (HF/OSDA^+^ > 1.0) led to the crystallization of polymorph A-enriched (55–65%) zeolite beta. Based on ^19^F MAS NMR analysis, they proposed that at high HF concentrations most F^–^ is converted to SiF_6_^2–^, forming an H^+^/F^–^ buffer that continuously supplies small amounts of fluoride during crystallization, thereby influencing polymorphic enrichment in beta zeolites.^97^ For germanates, Guo et al. showed that Ge_10_ clusters are preferentially formed under zero or low HF concentrations, whereas the formation of Ge_7_ clusters requires high HF concentrations.^98^

A gel composition with HF/OSDA^+^ = 1.0 has also been commonly employed for the synthesis of aluminosilicate zeolites. For example, aluminosilicate RTH and LTA zeolites (Si/Al > 7) have been synthesized via the fluoride route using various imidazolium-based OSDAs.^99–102^ In 2018, Jo et al. and Shin et al. reported the synthesis of two zeolites with novel framework structures, PST-21 (PWO) and PST-22 (PWW), using 1,2,3-trimethylimidazolium (123TMI^+^) and 1,2,3,4-tetramethylimidazolium (1234TMI^+^), respectively, under ‘excess fluoride’ conditions (HF/OSDA^+^ = 2.0, which can be generalized to HF/OSDA^*q*+^ = 2*q* for multicharge OSDAs).^77,103^ Under otherwise identical conditions but at the standard fluoride concentration (HF/OSDA^+^ = 1.0), both 123TMI^+^ and 1234TMI^+^ predominantly yielded RTH zeolite. Based on the premise that the concentration of F^–^ ions may influence product selectivity during aluminosilicate crystallization, and given that isomorphous substitution of Si by Al in the zeolite framework competes with F^–^ encapsulation in the crystallized products for charge balance with the OSDAs, they proposed that variations of HF concentration in the synthesis mixture could unlock previously unrealized structure-directing potentials of known OSDAs. The product selectivity outcomes from the excess fluoride approach are summarized chronologically in Table 1, and the OSDAs employed are given in Figure 5.

**Table 1:** Differences in the Zeolite Product Selectivity Under Standard and Excess Fluoride Conditions

		gel Si/Al ratio	zeolite product^b^				
year	OSDA^a^		standard conditions (HF/OSDA^*q*+^ = 2*q*)	excess conditions(HF/OSDA^*q*+^ > 2*q*)	Si/Al ratio^c^	feature	ref
2018	123TMI^+^	10	RTH^f^ (8, 2, 16.1)	PWO (9, 2, 17.1)	10	novel structure	103
2018	1234TMI^+^	10	RTH^f^ (8, 2, 16.1)	PWW (10, 2, 17.0)	11	novel structure	103
2018	choline^+^	15	RUT^f^ (8, 0, 18.1)	ESV (8, 1, 17.7)	14	–	110
2019	13DMP-C_4_^2+^	10	RTH^f^ (8, 2, 16.1)	PTY (10, 2, 17.0)	11	novel structure	104
2020	PMI^+^	∞	STW (10, 3, 16.4)	PST-24^g^ ^,^ ^i^ (10, 2, 18.7–18.8)	∞	novel structure	107
2021	C_3_MPp^+^	10	beta^f^ ^,^ ^g^ (12, 3, 15.1–15.3)	MWW^j^ (10, 2, 15.9)	–	–	113
2021	C_3_MPp^+^	20	beta^f^ ^,^ ^g^ (12, 3, 15.1–15.3)	EUO^i^ (10, 1, 17.1)	–	–	113
2022	123TEI^+^	4.0^d^	DON^h^ (14, 1, 17.1)	PTF (10, 2, 16.0)	3.9^d^	novel structure	109
2023	Me_4_Et_2_-diquat-6^2+^	50	amorphous	ITR (10, 3, 17.4)	52	–	112
2023	Me_4_Et_2_-diquat-6^2+^	15^e^	amorphous	ITR (10, 3, 17.4)	75^e^	–	112
2023	*i*P13DPI^+^	10	MEL^f^ (10, 3, 17.4)	RTH/ITE^g^ (8, 2, 15.7–16.1)	–	–	106
2025	134TMP^+^	10	FER^f^ (10, 2, 17.6)	PWO^i^ (9, 2, 17.1)	11.5	–	111

a See Figure 5.

b The numbers in parentheses are, from left to right, the largest ring size, topological (pore opening >6R) channel dimension, and FD_Si_.

c Determined by elemental analysis.

d Si/Ge ratio.

e Si/B ratio.

f Major phase in the product mixture.

g Disordered phase.

h Minor phase coexisting with PTF.

i HF/OSDA^+^ = 3.0.

j Referred as to the condensed 3D MWW structure.

**Figure 5:**
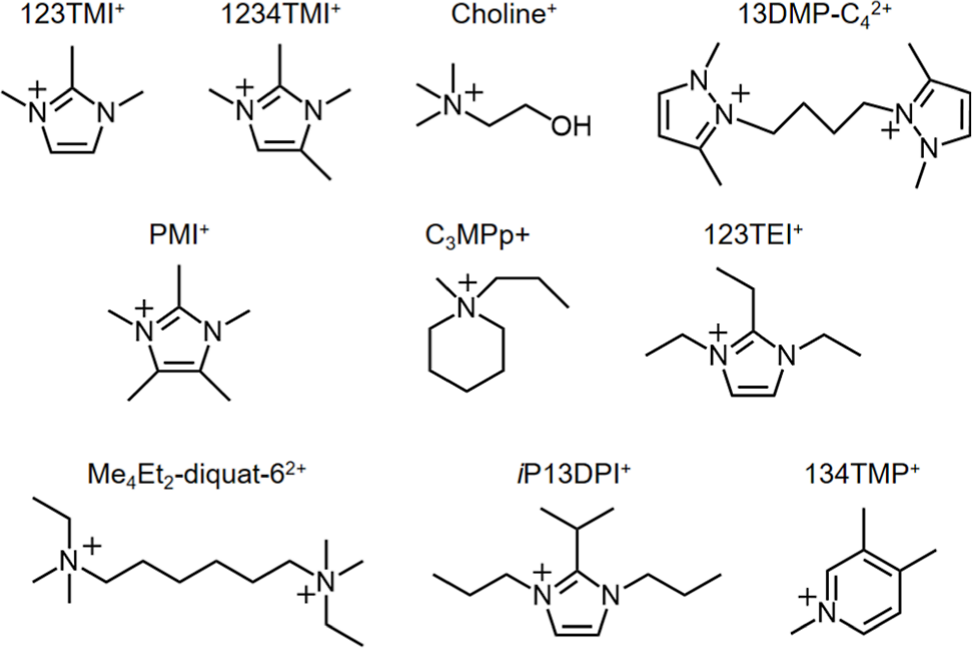
Some OSDAs studied under the excess fluoride synthesis conditions.

**Figure 6:**
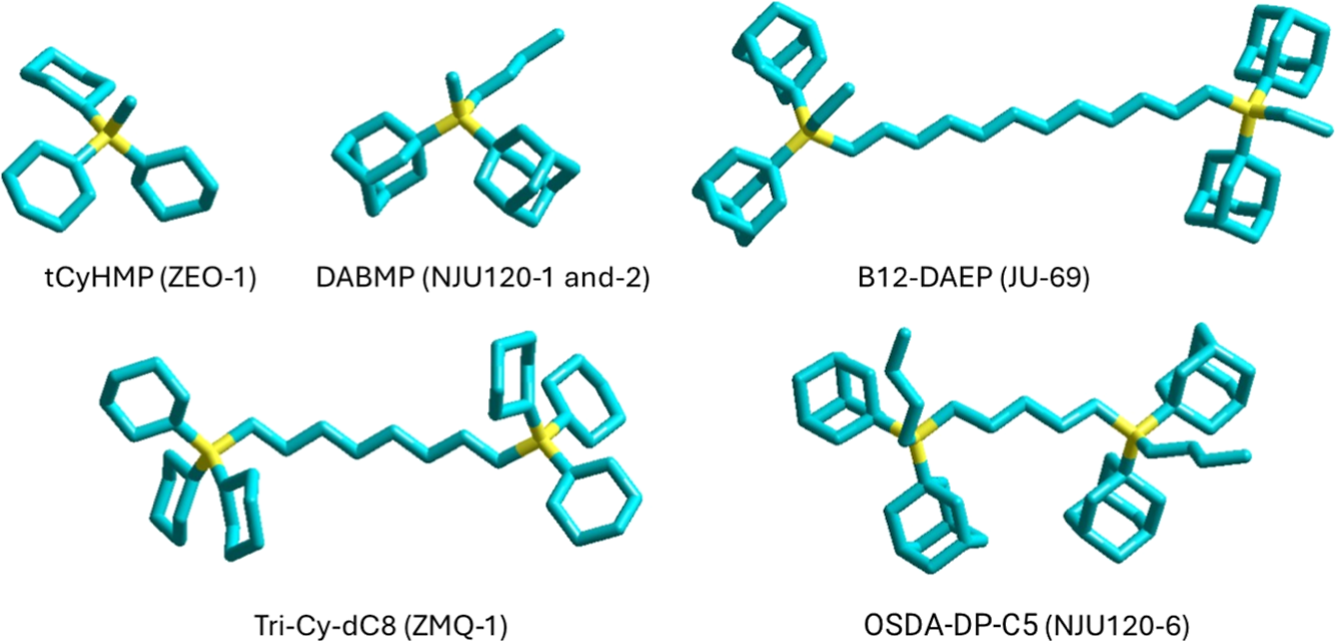
Phosphonium cations used for the synthesis of stable silica-based ultraporous zeolites (shown in parentheses). The acronyms from the original publications have been retained, and they refer to the following cations: tricyclohexyl­(methyl)­phosphonium (tCyHMP); di­(1-adamantyl)-*n*-butyl-methylphosphonium (DABMP); 1,12-bis­[di­(1-adamantyl)-ethylphosphonium]­dodecane (B12-DAEP); bis-1,8­(tricyclohexylphosphonium)­octamethylene (Tri-Cy-dC8); bis-1,5­(bis­(1-adamantyl)­butylphosphane)­pentane (OSDA-DP-C5).

Aluminosilicate PST-30 (PTY), featuring another novel framework structure, was synthesized using a pyrazolium-based dication, a member of a class of OSDAs that has received considerably less attention than imidazolium derivatives (HF/13DMP-C_4_^2+^ = 4.0).^104^ Notably, the PST-30 framework had been conceptually derived from structural insights of PST-21 and PST-22. This targeted synthesis was successfully realized by retaining the original PST-21/-22 synthesis protocol and replacing only the OSDA with a computationally predicted one, under the same excess fluoride conditions. Subsequently, the 13DMP-C_4_^2+^ dication was shown to direct the synthesis of PST-30 even in hydroxide media, whereas it failed to do so under standard fluoride conditions.^105^ In the same work, another pyrazolium-based dication was used to crystallize pure PST-22 in hydroxide media. On the other hand, 2-isopropyl-1,3-dipropylimidazolium directed the synthesis of a pure RTH/ITE intergrowth at OSDA^+^/SiO_2_ = 1.0 and HF/OSDA^+^ = 2.0, although RTH/ITE was already a major phase at lower OSDA^+^/SiO_2_ ratios or when other 2-isopropylimidazolium-based OSDAs were employed.^106^ Notably, a new type of MEL/MFI intergrowth along the [100] direction was also detected in trace amounts when using different 2-isopropylimidazolium-based OSDAs.

Beyond aluminosilicate zeolites, PST-24 is a new medium-pore pure-silica intergrowth with varying intracrystalline channel dimensionality synthesized via the excess fluoride approach, despite being unrelated to the original premise of this strategy which was based on the competition between Al-substitution and F^–^ encapsulation.^107^ Furthermore, this disordered zeolite requires even more extreme fluoride-excess conditions (HF/OSDA^+^ = 3.0) for the crystallization and exhibits 2D disorder, in which *d5r* column pairs can adopt two different arrangements that either close or open 10-ring channels. As a result, the local channel dimensionality can vary from 2D to 3D within a single crystal. Its aluminosilicate version (Si/Al = 47), synthesized using a seeding technique, was found to be an efficient catalyst for low-temperature isobutanol dehydration.^108^ Similar to the case of PST-24, PST-35 (PTF) does not contain trivalent T-atoms but instead incorporates Ge atoms.^109^ Even under excess fluoride conditions, Ge atoms still exhibits strong preference for forming *d4r*s.

The excess fluoride approach has not been limited to diazolium-based OSDAs and the discovery of novel framework structures. A high-silica (Si/Al = 14) version of zeolite ERS-7 (ESV) was also synthesized using choline ions as an OSDA at a gel HF/OSDA^+^ ratio of 2.0, demonstrating that the excess fluoride approach is a viable route to accessing new compositional domains.^110^ 1,3,4-trimethylpyridinium directed the crystallization of PST-21 with a slightly higher Si/Al ratio (11.5) under excess fluoride conditions, compared to PST-21 synthesized using 123TMI^+^ (Si/Al = 10), which was attributed to unexpectedly large amounts of SiO^–^···HOSi defects.^111^ A similar trend was observed for PWW and RTH zeolites (Si/Al ∼14) synthesized using different pyridinium-based OSDAs. In 2023, Ma et al. reported the synthesis of Ge-free ITR zeolite, a “twin” of the ITH framework (i.e., possessing a highly similar structure), using a diquaternary OSDA.^112^ This achievement was based on a detailed understanding of the structural similarities and differences between the ITH and ITR structures and was further supported by theoretical simulations. For aluminosilicate and borosilicate compositions, the excess fluoride approach was found to suppress the formation of an amorphous phase. Additionally, while certain *N*-alkyl-*N*-methylpiperidinium ions (e.g., C_3_MPp^+^) directed the formation of several zeolite phases under excess fluoride conditions, including EMM-10P (an MWW precursor) and EU-1 (EUO), *N,N*-dimethylpiperidinium enabled the crystallization of levyne (LEV) over a broad HF/OSDA^+^ range (0.5–2.0).^113^ Nevertheless, detailed characterization of these levyne samples revealed that the gel HF content plays a critical role in determining not only the Al distribution within the zeolite framework but also the crystal size.

To gain deeper insight into the structure-directing roles of Al and F^–^ in zeolite synthesis, Altundal and Sastre performed force field simulations on PST-21, PST-22, and ERS-7, which were synthesized via the excess fluoride approach.^114^ Their results demonstrated not only that the structures with the lowest calculated synthesis energies correspond to those experimentally obtained under standard fluoride conditions, but also that the new structures formed under excess fluoride conditions have slightly higher synthesis energies among the competing zeolite phases. It is worth noting that under excess fluoride conditions, the concentration of non-Si T-atoms in the zeolite products is generally comparable to that in their synthesis mixtures, although no general empirical trend has been established for the structures formed as a function of HF concentration so far (Table 1). Expanding the scope to a broader range of fluoride concentrations, more recent studies have shown that a ‘deficient fluoride approach’ (HF/OSDA^+^ ≤ 0.5) under moderately concentrated conditions (H_2_O/SiO_2_ ∼10) can accelerate zeolite crystallization and enable control of both the crystal size and defect concentration.^115,116^ We anticipate that fluoride concentration will remain a key parameter in zeolite synthesis and that the relationship between fluoride concentration and zeolite product formation will be further clarified.

### Stable Ultraporous Zeolites

Over the past few years, access to new stable silica-based zeolites with greatly improved porosity has been discovered and has started to flourish, making the realization of a long-standing goal in zeolite research. The pioneering zeolite is ZEO-1, the first stable, silica-based zeolite with a 3D system of ELPs with large supercages.^117^ For the purpose of this perspective, and to characterize a whole new class of materials, any zeolite with a framework density equal to or smaller than ZEO-1 (JZO; 13.2 T-atoms/1000 Å^3^) will be denoted here as “ultraporous”. In less than 5 years, seven ultraporous stable zeolites have been reported, showing the rapid growth of the field. All of their syntheses have in common the use of phosphonium cations as an OSDA at some stage, while two of them require additional legochemistry (Table 2 and Figure 5).

**Table 2:** Stable Ultraporous Silica-Based Zeolites

zeolite		synthesis^a^				structure				ref
	route	OSDA	*T* (°C)	T-atom	FD_Si_ ^b^	channel (R)	pore size^c^	*V*_pore_ ^d^	ZFT^e^	
ZEO-1	F^–^, OH^–^	tCyHMP	175–190	Si, Al	13.2	(16 + 12) × (16 + 12) × (16 + 12)	10.6 × 9.4,1, 0.5 × 9.6, 7.2 × 6.6, 7.2 × 5.5	0.35	JZO	^117^
ZEO-3	1D-to-3D topotactic condensation^f^			Si	12.8	16 × 14 × 14	10.4 × 8.5, 9.8 × 8.0	0.33	JZT	^122^
ZEO-5	interchain expansion^f^			Si	11.1	20 × 16 × 16	10.7 × 10.7, 11.5 × 8.0	0.39	HZF	^123^
NJU120–1	OH^–^	DABMP	175–190	Si, Al	12.3	22 × 10 × 10	15.5 × 12.3, 5.4 × 5.7	0.32	NJO	^127^
							5.3 × 5.8			
NJU120–2	OH^–^	DABMP	175–190	Si, Al	12.4	22 × 12 × 10	16.6 × 11.4, 7.6 × 5.7, 5.8 × 5.3	0.35	NJW	^127^
ZMQ-1	F^–^, OH^–^	Tri-Cy-dC8	180–190	Si, Al	11.4	28 × 10 × 10	22.8 × 11.8, 6.3 × 5.9	0.47	–	^125^
							5.6 × 5.6, 5.9 × 5.1			
NJU120–6^g^	F^–^, OH^–^	OSDA-DP-C5	175–200	Si, Al, Ti	9.4	36	25.7 × 19.1	0.66	–	^128^
JU-69^g^	OH^–^	B12-DAEP	175	Si	9.5	36	25.2 × 19.5	0.61	–	^129^

a Conditions in the reference reporting the discovery of the zeolite. For full OSDA names, please check the caption in Figure 6.

b Experimental framework density as number of T-atoms per 1000 Å^3^.

c Crystallographic pore dimensions in Å calculated from the distance between opposite framework oxygen atoms, after subtracting twice the van der Waals radius of oxygen (2.7 Å).

d Reported pore volume in cm^3^ g^–1^ obtained from N_2_ isotherms, except the one of NJU120–6, which was obtained from Ar measurements. Pore volume for ZEO-5 is given as the average of samples prepared from different conditions (see ref 117).

e Zeolite Framework Type code assigned by the Structure Commission of the International Zeolite Association. ″–″ means no code assigned yet.

f Nonhydrothermal synthesis.

g NJU120–6 and JU-69 have been found to possess the same structure.

ZEO-1 is a stable aluminosilicate zeolite synthesized via hydrothermal crystallization using tricyclohexylmethylphosphonium as an OSDA, and it possesses 3D interconnected (16 + 12)­R channels. Due to the relatively higher stability of phosphonium cations compared to ammonium OSDAs, it affords a higher zeolite synthesis temperature than its N-based counterparts.^118^ Synthesized at 190 °C in F^–^ or OH^–^ media, ZEO-1 exhibits high thermal and hydrothermal stability, even upon calcination at 1000 °C and steaming at 760 °C. Subsequent to calcination, extensive water washing effectively eliminated all residual P species remaining. Compared with the 12R supercages of FAU, JZO features three different types of large supercages, each possessing four 16R and/or 12R windows. With an extremely large specific surface area and excellent hydrothermal stability, this ELP zeolite exhibits superior performance in dye adsorption, highlighting its potential as a promising adsorbent for the removal of organic pollutants. In FCC, ZEO-1 outperforms the prototypical zeolite FAU used in this process. With more accessible Brönsted acid sites, ELP ZEO-1 has better performance in the carbonylation and disproportionation of dimethoxymethane.^119^ Based on its superior porosity, ZEO-1 was used as a template in the synthesis of zeolite-templated carbon (ZTC), forming ZTC-JZO, which exhibits high electrical conductivity.^120^ The coke-resisting behavior during propene transformation was also investigated, concluding that, due to its wider pore system, ZEO-1 shows improved resistance to coke poisoning than USY, despite larger coke formation in the former.^121^

On the other hand, when the synthesis is performed without Al using the same OSDA, ZEO-2, a chain silicate, was obtained instead of ZEO-1.^122^ An unprecedented 1D-to-3D topotactic condensation of ZEO-2 was achieved via calcination, resulting in the 3D ELP zeolite ZEO-3 (JZT) with 16 × 14 × 14R channels. Similar to ZEO-1, ZEO-3 shows robust thermal and hydrothermal stability, which was confirmed by the well-preserved PXRD patterns after calcination and steaming. Ti-ZEO-2 was hydrothermally synthesized directly from a gel with the addition of tetrabutylorthotitanate as a titanium source. Calcination under H_2_ atmosphere enables the condensation of Ti-ZEO-2 to form Ti-ZEO-3. The high specific surface area and 3D channel system of ZEO-3 allow the diffusion and adsorption of large molecules, such as Nile Blue, outperforming the capacity of zeolite beta and demonstrating its capacity for decontamination. Furthermore, ZEO-3 also adsorbs toluene even in the presence of water and shows a lower recovery temperature than beta, highlighting its potential for volatile organic compounds abatement and recovery.

Shortly thereafter, ZEO-2 was employed in a pioneering interchain expansion reaction to synthesize the interrupted ZEO-4A or ZEO-4B frameworks using silane reagents (dimethyldichlosilane, i.e., DCDMS or 2,4,6,8-tetramethylcyclotetrasiloxane, i.e., s4r, respectively) in HCl–EtOH solution.^123^ The OSDA within the ZEO-2 framework was removed intact during interchain expansion, which can enable OSDA recovery. ZEO-2 chains are connected by D^2^ or T^3^ Si atoms, i.e., (SiOSi)_2_ SiR_2_ and (SiOSi)_3_ SiR units, depending on their respective silane reagents, leading to the formation of *lau* units in ZEO-4A and *d6r* units in ZEO-4B. Upon calcination, both interrupted zeolites ZEO-4A and ZEO-4B transform by topotactic condensation into a new fully connected framework ZEO-5 (HZF) upon removing methyl groups and condensing adjacent Si–OH groups. Both *lau* and *d6r* units in ZEO-4 convert into unprecedented triple 4R (*t4r*) units in ZEO-5. Pure-silica ZEO-5 contains a 3D system of 20 × 16 × 16R pores, and a remarkably high surface area. Combined with its outstanding thermal and hydrothermal stability, these features pave the way for its applications in catalysis and adsorption. ZEO-5 is able to adsorb more toluene than beta, ZEO-3, and ZEO-4. Ti was introduced into the ZEO-5 framework using TiCl_4_ vapor treatment, producing a promising catalyst for the epoxidation of propylene to propylene oxide via the selective cumene approach. Very recently, it was shown that adsorption of water in ZEO-5 can break some bonds in *t4r* and lead to an apparent structure degradation in a process that is, however, fully reversible.^124^ This is related to the highly stressed nature of *t4r*s, evidenced by the highly distorted geometry around the Si atoms in the central *4r*, which produced a ^29^Si MAS NMR Q^4^ signal at the exceptional low chemical shift of −98 ppm.

Recently, the zeolite pore size has been extended into the quasi-mesoporous and even mesoporous regimes. ZMQ-1 is the first aluminosilicate zeolite with a meso-microporous channel system (28 × 10 × 10R), which can be synthesized in hydroxide and fluoride media, using bis-1,8 (tricyclohexylphosphonium) octamethylene as an OSDA.^125^ This zeolite has excellent thermal and hydrothermal stability up to 800 °C, and its thermal stability is enhanced further (stable up to 1000 °C) after calcination and ammonium exchange. Interestingly, the terminal silanol (SiOH) groups of the as-made ZMQ-1 interrupted framework are fully connected after calcination, transforming the bilobal-shaped 28R channel into an elliptical one. In vacuum gas oil (VGO) cracking tests, ZMQ-1 shows a higher conversion than MCM-41, while comparable to USY and beta. Besides, ZMQ-1 is a potential candidate as a scaffold for CO_2_ hydrate formation.^126^

Using di­(1-adamantyl)-*n*-butyl-methylphosphonium (DABMP) as the OSDA, ultraporous aluminosilicate zeolites NJU120–1 (NJO) and NJU120–2 (NJW) were crystallized in hydroxide media with different Al fractions and water contents.^127^ Both zeolites maintain their structural integrity after calcination at 1000 °C and steaming at 800 °C. The remaining P species within the zeolite frameworks after calcination were removed by ion exchange with NH_4_Cl solution. NJU120–1 and NJU120–2 feature interconnected channels with 22 × 10 × 10R and 22 × 12 × 10R pores, respectively. These zeolites both have straight 22R channels along one direction, providing a large diameter of channels (up to around 16 Å) for molecular diffusion. They were also tested as FCC catalysts, showing higher cracking efficiency for VGO than P-free ZEO-1, commercial USY and beta. A fully connected ultraporous zeolite family (NJU120–1-G*n*) was proposed through modulating the layer gliding and the number of interlayer pillars, showing ZQM-1 as one of its members.

Finally, by the time of writing this article, two bulky phosphorus-based diquaternary OSDAs, bis-1,4­(bis­(1-adamantyl)-butylphosphane)­butane (OSDA-DP-C4) and bis-1,5­(bis­(1-adamantyl)-butylphosphane)­pentane (OSDA-DP-C5) were used in the discovery of the ultraporous NJU-120–6.^128^ Only OSDA-DP-C5 afforded the material in pure form. NJU-120–6 is a mesoporous zeolite with 1D 36R channels and an interrupted framework. Adjacent 36R layers in NJU-120–6 are connected by Si–O-T linkages, resulting in relatively dense pore walls. However, the pore walls contain single 6R windows and, since the OSDA can be removed through the main mesoporous channel, these are very likely ultrasmall open pores. Despite its interrupted framework, NJU-120–6 maintains its crystallinity after calcination up to 900 °C, indicating outstanding thermal stability. The material can be synthesized not only as an aluminosilicate exhibiting enhanced performance in VGO FCC, but also as a titanosilicate with very good performance in the epoxidation of cyclohexene. It should be noted that JU-69, synthesized using a different phosphonium dication, 1,12-bis­[di­(1-adamantyl)­ethyl phosphonium]­dodecane and reported shortly after NJU-120–6, possesses the same structure as NJU-120–6.^129^ The excellent stability and the dangling SiOH groups within the 36R channels in JU-69 were reported to allow the anchoring of noble metals: Pd-loaded JU-69 has higher pyrene conversion and hydrogenation product selectivity compared with Pd-loaded MCM-41 and beta. NJU-120–6 and JU-69 are the most open zeolite reported to date and yet they remain highly stable, indicating that the upper limit of stability for very open zeolites has not yet been reached. Their appearance in such close temporal proximity, together with the reporting of all seven stable ultraporous zeolites in less than five years, highlights the emerging character of the field of ultraporous materials synthesized using phosphonium cations. However, although the field of ultraporous zeolites is growing fast, ZEO-1, ZEO-3 and ZEO-5 continue to be the only fully connected, silica-based zeolites with 3D systems of extra-large pores. As shown in Table 2, the synthesis of all stable ultraporous zeolites has required at some point the use of phosphonium cations at relatively high temperature and very likely further advances in porosity will follow the same trend. Finally, we want to point out that all ultraporous zeolites in Table 2 have contradicted the long-standing claim of Brunner and Meier that the minimum framework density increases with the size of the smallest rings in the network.^130^ This idea, which had been held for more than three decades, was considered to have a predictive value that could guide efforts to synthesize zeolites of lower density.

## Current Challenges in Zeolite Synthesis

While the ability of both conventional and unconventional pathways to discover new zeolites is beyond dispute, significant challenges remain for the mature widespread commercial application of most known zeolites, especially regarding scalability, reproducibility, sustainability, and affordability. Conventional batch hydrothermal synthesis faces difficulties under industrially relevant conditions due to typically long crystallization times, thermal gradients in large autoclaves, and uneven heat distribution, which may hinder uniform crystal growth and compromise reproducibility. Nonconventional methods, such as continuous-flow reactors,^131^ may offer faster crystallization (down to minutes) and higher space-time yields, but they introduce new obstacles in reactor design and industrial upscaling. Zeolite production is highly energy-intensive: crystallization alone can generate well above 1 kg of CO_2_ per kg of zeolite,^132^ to which the energy consumed in drying, calcination and wastewater treatment must be added. Conventional routes generate hazardous emissions (NO_*x*_ and CO_*x*_) during OSDA calcination and produce considerable liquid waste, whereas unconventional solvent-free pathways reduce these impacts but may still rely on fluorides or high-temperature processes.

High production costs arise mainly from expensive OSDAs (quaternary ammonium or phosphonium cations, imidazolium cations and others), energy consumption, and high-purity raw materials. Commercial analyses report that energy can account for about 20–30% of total production costs, and specialty synthetic zeolites produced by template-assisted routes can exceed 1500 USD per ton.^133^ Hydrothermal methods further amplify costs through waste handling and relatively low yields, whereas unconventional OSDA-free or mechanochemical routes have the potential to lower costs by avoiding calcination and organic solvents.^134^

OSDA sustainability can be addressed through a number of strategies. First, degradable structure-directing agents that can be cleaved and removed under milder conditions afford the preparation of zeolites with open pores without recurring to high calcination temperatures, lowering energy consumption and the emission of pollutants.^135^ Second, recycling of OSDA-containing mother liquors reduce both OSDA use and wastewater generation.^136^ And third, more sustainable zeolite synthesis is expected to rely increasingly on OSDA- and solvent-free, near-neutral, seed-directed, and combined routes, and some advances have been achieved toward reducing OSDA consumption and waste generation.^137^ However, these approaches remain effective only for a limited set of selected zeolite types, and most frequently for high-Al materials, although it may be argued that the truly green synthesis of zeolites is still in its infancy. For example, an OSDA-free synthesis of ZSM-5 based on mechanochemical mixing of dry reagents and a small amount of water successfully produced batches up to 120 g in 200 mL autoclaves with uniform crystallinity and very high yield.^134^ Nevertheless, the relatively high synthesis temperature (200 °C), still long crystallization time for an industrial process (>24 h) and the narrow compositional window limit the broader benefits of the approach, which can be presumed to be difficult to extend straightforwardly to many other zeolite systems, especially the unconventional ones described in this Perspective. In this context, we would like to emphasize that the discovery of new zeolite frameworks is not an end in itself but a means to tailor pore architecture, composition, and stability to specific process constraints, thereby improving throughput, selectivity, and lifetime in industrial applications. The added value of further structures will thus depend on their ability to offer clear performance or sustainability advantages over existing materials—such as lower operating temperatures, higher regeneration efficiency, or compatibility with greener synthesis routes—rather than merely enlarging the structural catalogue. Ultimately, the success of new zeolites will depend on their relevance and industrial competitiveness. Suitable applications focusing on the selectivity that the new porosities can offer would eventually fuel new synthesis routes and/or new industrial developments to overcome the difficulties presented here, as it has been the case with commercial zeolites like, for instance, ZSM-5, Beta, Mordenite or Ferrierite.

## Future Perspectives

Given that it has been the case for the last about eight decades, it is reasonable to expect that new research in zeolite synthesis will keep expanding the number of available zeolite framework types, likely with a stronger emphasis on the highly open, low-density end of the spectrum, as suggested by Figure 1 and the very recent and accelerated discovery of ultraporous zeolites. In particular, the combination of ELPs with channel multidimensionality shows that framework density of stable zeolites can still be pushed downward, while maintaining crystallographic order, even if the pool of truly robust ELP (alumino)­silicates still remains relatively small. In this respect, one can wonder if there is an intrinsic limit to the porosity of stable zeolites (in terms of total density and/or of pore size).

The use of phosphonium cations has been the driving force behind the discovery of ultraporous zeolites. Their stability in the synthesis medium, especially in OH^–^ media, together with the possibility of preparing bulky organic cations, has enabled the emergence of this new class of zeolites (Table 2 and Figure 7). Since no limitations in either feasibility or stability have been observed for the materials obtained so far, we anticipate that the conventional synthetic approach will continue to be explored using phosphonium cations, as quaternary ammonium cations have been employed over the past 60 years. In practice, this involves screening synthesis conditions that combine bulky, rigid phosphonium OSDAs with systematic variation of key parameters like the type of mineralizing agent (OH^–^ or F^–^), the water content (preferably at low H_2_O/Si ratios), and the temperature and duration of the synthesis. The high stability of these cations allows the exploration of broader ranges of both temperature and water content than those typically accessible with ammonium-based OSDAs. Both the “multiple inorganic cation” and “excess fluoride” approaches are also expected to contribute to the exploration of synthesis conditions in phosphonium-based OSDA systems. It is difficult to know to what extent new unconventional routes will drive this expansion, but they will almost certainly contribute significantly, motivated by the appeal of accessing “unfeasible zeolites” and by sustainability constraints. Solid–state or solid-like conversions, 1D and 2D to 3D topotactic condensations, 1D and 2D expansions, the ADOR approach, and intergrowth engineering have already delivered frameworks that would have been very hard to obtain by classical hydrothermal OSDA chemistry alone. Various OSDA- and/or solvent-free routes have also contributed to more sustainable and affordable synthesis. In the future, these routes will likely be evaluated not only by the novelty of the topologies they deliver, but also by their compatibility with large-scale processing (continuous flow, solid-state reactors) and with more environmentally friendly options for raw materials, OSDAs (or lack of), and energy saving.

**Figure 7:**
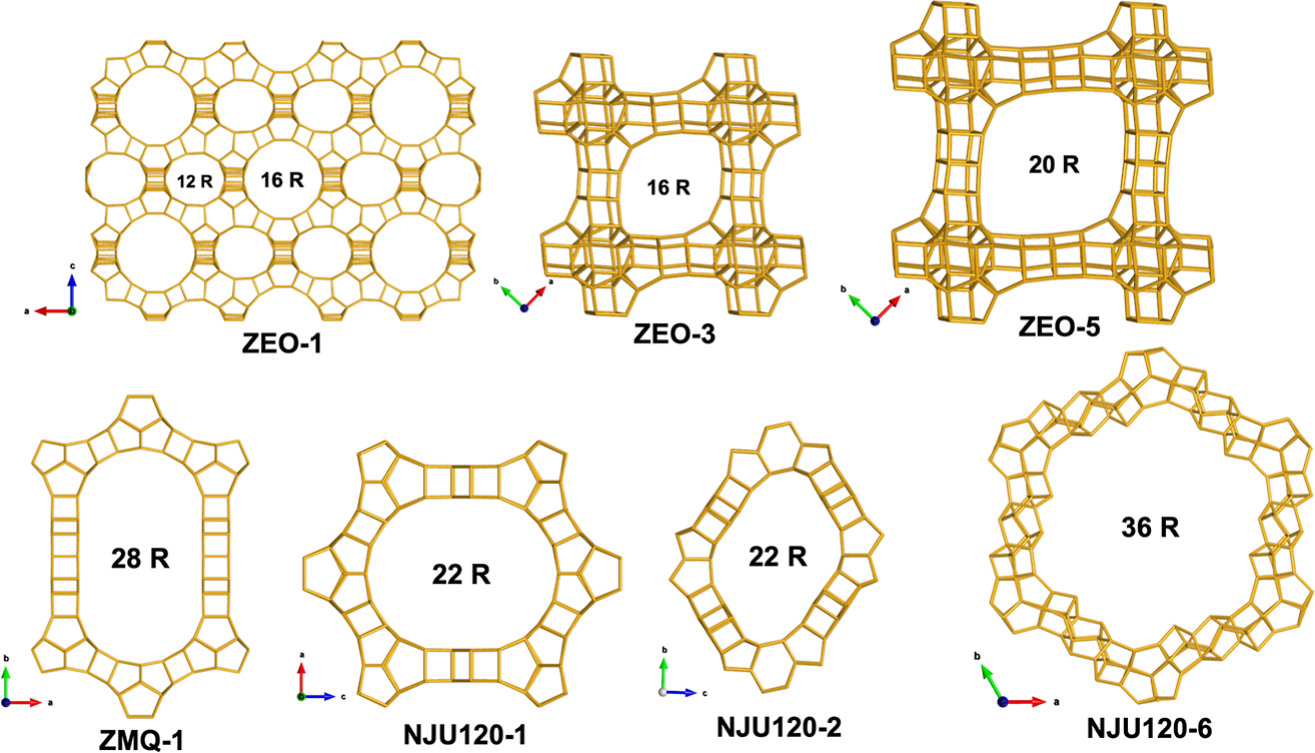
Seven stable ultraporous zeolite structures known to date.

Beyond the established unconventional routes discussed in this article, some emerging structure-direction concepts based on metal–organic coordination chemistry offer additional strategies for zeolite discovery. As very recently reported, metal-amine coordination polymers can serve dual roles as structure-directing agents and as source of inorganic framework components through metal–oxygen–silicon linkages. This approach is exemplified by the synthesis of inorganic–organic hybrid organozincosilicate LEU-1, where 1D Zn-amine coordination polymers extend 2D siliceous layers into 3D porous architectures. Furthermore, calcination of LEU-1 leads to a new silicate zeolite, LEU-2, with a new framework type (KLW). Given the versatility of metal-amine chemistry and of their geometries, this strategy opens up new avenues for obtaining new zeolite topologies.^138^

The very recent and rapidly growing field of artificial intelligence (AI) is already in its infancy in its application to zeolite synthesis but we can expect important developments in this field in the coming years.^88^ AI combined with big data mining of both successful and failed experiments (which may be less accessible because of the current trends in scientific publishing), and accurate descriptors of the energy of the synthesis, should result in guided experiments aimed toward particular structures, compositions or porosity motifs, departing from the current large screening of experimental variables. However, it has been recently shown that this objective may still not be reached because of the requirement of much improvement in computational methods.^139^ AI could also propose both hypothetical frameworks and corresponding synthesis windows, narrowing the gap between digital design and laboratory realization. However, the very complex nature of zeolite crystallization makes difficult to extrapolate across broader compositional spaces, to account for (alumino)­silicate solution chemistry, to avoid local minima and kinetic traps, or to deal with nucleation, phase transformation, and competing intergrowth formation. Machine learning may also be of interest in determining potential applications of a given zeolite structure type, especially if negative results could be as accessible as positive ones currently are.^140^
